# New Hope for Genome Editing in Cultivated Grasses: CRISPR Variants and Application

**DOI:** 10.3389/fgene.2022.866121

**Published:** 2022-07-18

**Authors:** Asad Riaz, Farah Kanwal, Iqrar Ahmad, Shakeel Ahmad, Ayesha Farooq, Claus Krogh Madsen, Henrik Brinch-Pedersen, Zelalem Eshetu Bekalu, Fei Dai, Guoping Zhang, Ahmad M. Alqudah

**Affiliations:** ^1^ College of Agriculture and Biotechnology, Zhejiang University, Hangzhou, China; ^2^ Centre for Advanced Studies in Agriculture and Food Security, University of Agriculture Faisalabad, Faisalabad, Pakistan; ^3^ Department of Agroecology, Research Center Flakkebjerg, Aarhus University, Slagelse, Denmark

**Keywords:** plant genome editing, CRISPR, Cas9, Cas12, SPRY, cultivated-grasses, stress tolerance

## Abstract

With the advent of Clustered Regularly Interspaced Short Palindromic Repeats (CRISPR) and CRISPR-associated protein (Cas) mediated genome editing, crop improvement has progressed significantly in recent years. In this genome editing tool, CRISPR-associated Cas nucleases are restricted to their target of DNA by their preferred protospacer adjacent motifs (PAMs). A number of CRISPR-Cas variants have been developed e.g. CRISPR-Cas9, -Cas12a and -Cas12b, with different PAM requirements. In this mini-review, we briefly explain the components of the CRISPR-based genome editing tool for crop improvement. Moreover, we intend to highlight the information on the latest development and breakthrough in CRISPR technology, with a focus on a comparison of major variants (CRISPR-Cas9, -Cas12a, and -Cas12b) to the newly developed CRISPR-SpRY that have nearly PAM-less genome editing ability. Additionally, we briefly explain the application of CRISPR technology in the improvement of cultivated grasses with regard to biotic and abiotic stress tolerance as well as improving the quality and yield.

## Introduction

Since the advent of agriculture, plants have been cultivated and utilized as a source of food and energy to feed humans and livestock. The world’s population is expected to increase by 40% in 2050 and the demand for food will be increased by 50% (Tilman et al., 2011; van Dijk et al., 2021). To meet the demand for food, crop production needs to be significantly improved in the near coming decades (Ronald 2011). With time, the evolutionary drive, domestication, and breeding of cultivated plants have transformed due to scientific advancement and environmental conditions. However, there is the utmost need for future crop improvement with better adapted to harsh weather and enhanced agronomical traits of yield (Koeppel et al., 2019).

Previously, the crops were improved by conventional breeding methods which are time-consuming and laborious. Those traditional methods are reinforced by modern molecular and genomic-based breeding techniques to meet future food demand (Riaz et al., 2021). Recently, molecular biology has progressed with several great discoveries including genome sequencing and genetic engineering. Many molecular approaches were introduced for genome editing which relied on site-specific recognition of DNA sequences via zinc finger nucleases (ZFNs) and transcription activator-like effector nucleases (TALENS) which rely on DNA-protein interaction and require case-by-case protein re-design (Urnov et al., 2010; Miller et al., 2011). The discovery of the bacterial Clustered Regularly Interspaced Short Palindromic Repeats (CRISPR) and CRISPR associated protein (Cas) system (Gasiunas et al., 2012; Jinek et al., 2012) and its adaptation to genome editing in various organisms including plants provided a much more accessible method that has initiated a revolution in crop improvement (Shan et al., 2013). A wide range of plants including agronomic crops such as rice, wheat, maize, and barley are being subjected to molecular improvement using the new breeding technology CRISPR, which facilitates genome editing by gene deletion or/and insertion, and replacement (Liu HJ. et al., 2020; Usman et al., 2020; Han et al., 2021; Zhang et al., 2021). The CRISPR-Cas 9 system was originally discovered in bacteria and archaea immune systems where it detects and degrades invasive DNA from bacteriophages and plasmids. The active component is a ribonucleoprotein complex consisting of a Cas RNA-guided endonuclease and guide RNA that recognizes target DNA through the variable protospacer motif (Fineran and Charpentier 2012). In order to effectively introduce double-strand breaks, CRISPR requires a PAM (protospacer adjacent motif) sequence to be present in the target DNA adjacent to the protospacer complementary sequence. This poses a limitation to genome editing designs and has motivated the search for variants of CRISPR tools with alternative PAM requirements. PAM is a short 2–6 bp sequence preceded by the targeted DNA sequence. Cas9 nuclease from the type II CRISPR-Cas 9 system of *Streptococcus pyogenes* is the most commonly used system and it requires an NGG (N, any nucleotide; G, guanine) PAM sequence for DNA targeting (Jinek et al., 2012). The CRISPR-based genome editing tool is now extensively embraced with a low-cost, fast, and easy-to-use targeted gene editing system to cultivated grasses (Cram et al., 2019; Kim et al., 2019; Zhang et al., 2020; Lawrenson et al., 2021). New variants of CRISPR with different PAM sequences such as CRISPR-Cas 12a (formerly known as Cpf1) (Chen et al., 2018) and CRISPR-Cas 12b (formerly known as C2c1) (Ming et al., 2020) have been developed. At the beginning of the year 2021, the application of the next generation of CRISPR-Cas variant known as CRISPR-SpRY has been developed without any PAM restriction that improves gene editing resolution (Walton et al., 2020). The progress in CRISPR technology increases the efficacy and specificity which significant improvement editing outcomes and widen the applications in crop improvement.

This review discusses the components of CRISPR-based genome editing tools in detail and presents a quick comparison of previous main CRISPR variants with the latest one based on PAM sequence requirements. Moreover, an overview of different applications of CRISPR-based genome editing tools is also covered in a schematic diagram and its application for the improvement of agronomic traits as well as biotic and abiotic stresses in cultivated grasses such as rice (*Oryzae sativa*), maize (*Zea mays*), barley (*Hordeum vulgare*) and wheat (*Triticum aestivum*) is explained briefly. Therefore, this review showed the application and usefulness of genome editing as a new breeding technology for crop improvement.

## CRISPR-Based Genome Editing Tool: Components and Mechanism

### Components

CRISPR/Cas9 has been widely studied, well understood, and extensively used (Liu ZQ. et al., 2020). Two major components are essential for typical engineered CRISPR-Cas systems, a Cas endonuclease protein, and a single guide RNA (sgRNA) of 20 nucleotide sequences that guide the Cas enzyme to the target sequence for introducing the double-stranded break (DSB) (Mahfouz et al., 2014). Multiple variants of Cas9 and gRNA are available according to their novel application in the field of genetic engineering in plants (Chen et al., 2019; Li et al., 2019).

Cas-9 consists of two regions, called the recognition (REC) lobe and the nuclease (NUC) lobe. The REC lobe has two multi-helix domains, named REC1 and REC2, essential components to bind with the guide RNA and target DNA (Makarova et al., 2017). REC1 is comprised of an extended α-helical structure of 25 alpha helices and 2 β-sheets, whereas REC2 has a six-helix structure and is embedded within the REC1 domain (Nishimasu et al., 2014). The NUC lobe has three - domains: RuvC, HNH, and PAM-interacting domains. In order to cut a double-stranded DNA, the REC lobe initiates the binding of sgRNA and DNA, whereas the RuvC and HNH domains properly execute the cleavage of the complementary and non-complementary strand of the target DNA, respectively. In the meantime, the carboxy-terminal residing PAM-interacting domain confers PAM interaction and specificity to the target DNA (Yamano et al., 2016).

Guide RNA is composed of two elements, CRISPR RNA (crRNA) and trans-activating CRISPR RNA (tracrRNA). The crRNA is a long sequence of 18–20 base pairs that recognize and specify the target DNA by binding with it (Barrangou 2013). The tracrRNA is a long, twisted structure that serves as a binding scaffold for Cas-9 nuclease (Li 2015). The sequence of tracrRNA is partially complementary to a segment of crRNA. And, the base pairing of complementary sequences results in an RNA-duplex (tracrRNA–crRNA) and activates the Cas9 to form the Cas9-crRNA-tracrRNA editing complex (Mojica et al., 2009). The structural engineering of tracrRNA–crRNA duplex into a single guide RNA (sgRNA) creates a dual component Cas9-sgRNA system that simplifies the editing of genomic regions (Jinek et al., 2012).

### Mechanism

Biological systems of CRISPR/Cas are a part of the adaptive immune system of bacteria and archaea, protecting them from nucleic acid invaders such as viruses by cleaving the alien DNA in a sequence-dependent fashion (Bortesi and Fischer, 2015). At the proximal end of a CRISPR locus, short fragments of the invading DNA (spacers) are integrated between two adjacent repeats to confer immunity to the invading cells. Upon subsequent encounters with invasive DNA, the CRISPR arrays and spacers are transcribed to produce the 40 nt small interfering crRNAs, which bind together with tracrRNAs to activate and guide the Cas9 nuclease (Karvelis et al., 2013). Cas9 activation tempts the REC lobe to undertake conformational changes and forms a central channel to accommodate the negatively charged guide-RNA and target-DNA heteroduplex in a positively charged interface between the REC and NUC lobes (Nishimasu et al., 2014). The RuvC and the PAM-interacting domain create a positively charged surface to interact with the 3′end of the sgRNA, and the catalytic domain HNH comes closer to the DNA cleavage site.

Upon finding the appropriate PAM for the target site, Cas-9 triggers local DNA melting followed by hybridization of RNA with DNA leading to the activation of Cas-9 protein to cleave target DNA (Jiang and Doudna, 2017). This process eventually results in the cleavage of homologous double-stranded DNA sequences known as protospacers in the invading DNA (Barrangou et al., 2007). Usually, the 5′-NGG-3′ PAM sequence is more preferred and frequently used than 5′-NAG-3’ (Hsu et al., 2013).

After DSB, the cellular DNA repair pathways commence. The repair mechanism induces the cell to undertake homology-directed repair (HDR), microhomology-mediated end joining (MMEJ) or non-homologous end joining (NHEJ). HDR occurrence leads towards specific editing by a repair template-specific desired genomic modification (Vu et al., 2017). HDR-based strategies have proven difficult in plants because of a high preference for the NHEJ pathway. Various strategies to overcome this plant-specific limitation have been proposed (Vu et al., 2020). MMEJ/NHEJ is an error-prone repair system that involves the arrangement of micro homologous sequences internal to broken ends prior to joining and is coupled with insertions and deletions (Afzal et al., 2020). Polymerase theta-mediated end joining (TMEJ) is an advanced form of MMEJ/NHEJ, used as break repair with the homology of >1bp sequences (Schimmel et al., 2019). In the case of NHEJ, no DNA repair template is provided, and its error-prone nature often leads to inactivating mutations such as small deletions (Trenner and Sartori, 2019). In the case of a perfect repair, the target may simply undergo a new cycle of DSB and repair. Some other repair mechanisms also exist like the single-stranded annealing (SSA) pathway of HDR, which requires only a single DNA duplex and uses the repeat sequences as the identical sequences as in HDR (Cejka and Symington, 2021). Thus, all these methods are efficient tools for genome editing that might be insertion/deletion, replacement or knockout of desired genes in the cultivated grasses for improvement.

## CRISPR-Variants: Old vs. Latest

**FIGURE 1:**
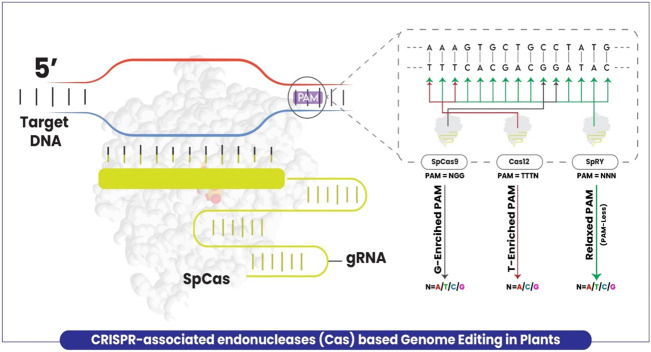
A schematic model explaining the plant genome editing using CRISPR-associated enzymes with limitations and extra feature of SpRY as compared to others. Cas9 targets a G enriched site with PAM = NGG (Black arrow), Cas12 targets T enriched site with PAM = TTTV (Red arrow), whereas SpRY with no PAM restriction (Green arrow).

Target specificity is provided by the base complementarity of the protospacer motif of the guide RNA. However, the DNA region targeted for cleavage by the enzyme has to be followed by the appropriate PAM sequence. The prototypical Cas9 derived from *Streptococcus pyogenes* (SpCas9) needs a GC-enriched site PAM in the form of NGG (N = A/T/G/C) which limits the targeting flexibility. The presence of PAM restrains access to some potential sites due to which many precision edits of targeting sites encoding non-canonical PAM remained inaccessible (Figure 1). Notwithstanding, several efforts were made to increase the flexibility of target site recognition by Cas enzymes (Nishimasu et al., 2018; Miller et al., 2020), and new endonuclease enzyme variants were developed such as Cas12a (Chen et al., 2018) and Cas12b (Ming et al., 2020). Similar to Cas9, these variants also retain the limitation of PAM requirement by relying on T-enriched at the 5′-end of PAM in the form of TTTV (V = A/G/C) (Figure 1). Recently, Walton et al. (2020) successfully overcame the limitation by developing the structure-guided engineered variant of the SpCas9 enzyme, named SpRY targeting the genomic DNA with the independence of PAM restriction (nearly PAM-less qualities) (NRN > NYN, where R is A or G and where Y is C or T) in human cells. Very recently, Ren et al. (2021) explored the versatility of this improved genome-editing tool in plants for the first time and proved that SpRY targeted a total of 59 NNN PAM sites (NAN/NGN/NCN/NTN) in rice. Cas9 was demonstrated for being unable to edit relaxed PAM sites and less efficient in non-canonical PAM sites than SpRY, which possibly achieved larger deletions up to five base pairs at relaxed PAM sites which is impossible using Cas9. Elimination of the PAM requirement exposes the CRISPR-Cas T-DNA to self-editing thus introducing a risk of gRNA inactivation or modification (Ren et al., 2021).

Likewise, single base editing using a CRISPR-mediated genome editing tool has also been developed and applied in different cultivated grasses. For example, cytosine base editing (CBE) and Adenine Base editing (ABE) have been optimized for base editing in rice, wheat, and maize (Shimatani et al., 2017; Zong et al., 2017; Li C. et al., 2018; Zong et al., 2018). But, these were inefficient at some targets and several strategies have been used to enhance their efficiency monocots. Fortunately, it was proved that the SpRY-PmCDA1 (PAM-less C to T nucleotide editor) successfully converted the C-to-T base in rice (Ren et al., 2021). Thus, the expanded target range of this CRISPR-associated–SpRY enzyme harnessed the high accuracy of base (nucleotide-level) editing by using SpRY-based cytosine base editors (CBEs) in relaxed PAM (first to sixth base of protospacer). This was not possible in the traditional C-to-T base editors due to the specific distance requirement in the editing windows (Manghwar et al., 2019). On the other hand, the SpRY-based adenine base editor (ABE8e) also showed higher efficiency of A-to-G conversion with an editing window of fourth to eighth bases of the protospacer (Ren et al., 2021). Hence, a novel choice of base number edits is now possible in plants using SpRY-based CBEs and ABEs. In the toolbox of a CRISPR-based system, PAMs play a vital role as a specific uniform for Cas enzymes by differentiating them from non-self DNA sequences (Westra et al., 2013). Therefore, the application of the SpRY base CRISPR tool for PAM-less targeting raised a significant limitation of self-editing, which was suggested to utilize for secondary off-targeting of novel and unaccounted edits. The only one identified off-target in multiple T0 transgenic rice lines could be taken as promiscuity of *de novo* spacer from a self-targeting gRNA vector (Ren et al., 2021).

These outcomes and unclear shortcomings compel further investigation of structural engineering and application in different systems notably such self-editing was not reported/observed for single base editing in human cells using SpRY-ABEs (Walton et al., 2020).

## Application of CRISPR Technology in Cultivated Grasses

**FIGURE 2:**
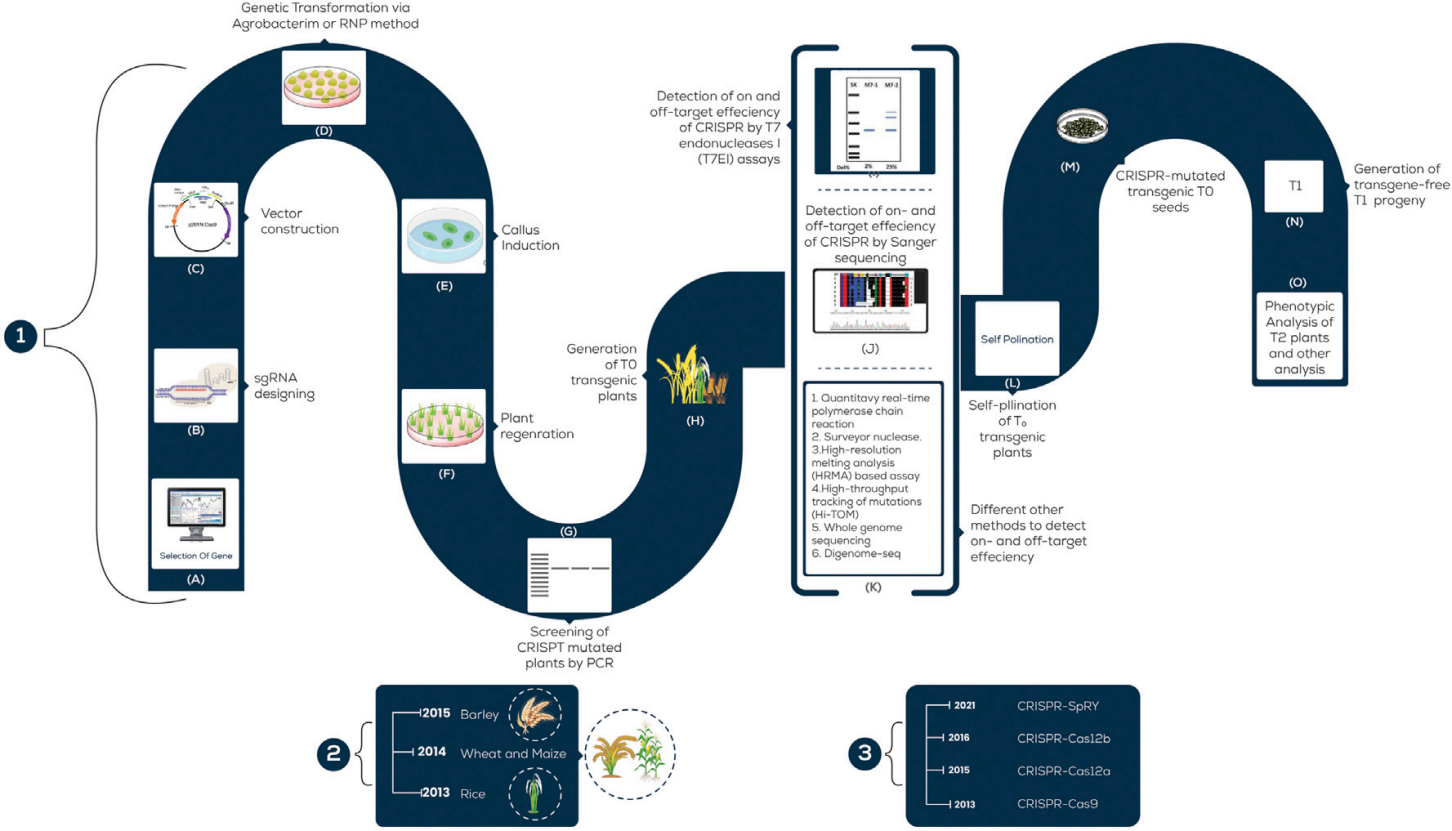
A schematic illustration of the steps involved in CRISPR/Cas9 Genetic Transformation; 1; **(A)** Specific gene-targeted; **(B)** Designing sgRNA for the desired gene; **(C)** Vector; **(D)** Transformation of the CRISPR/Cas9 system; **(E)** Callus formation; **(F)** Regeneration of shoots from callus; **(G)** T0- Mutated plants; **(H)** Transgenic plants testing by PCR; **(I)** Identification of mutated plants by T7E1; **(J)** Screening of mutants by sequencing; **(K)** Various techniques to detect edited plants; **(L)** Self-pollination of T0 transgenic plants; **(M)** Mutated T0 seeds; **(N)** T1 progeny; **(O)** Phenotypic analysis of T2 plants. (2) The scale mentions the year in which each grass was employed for CRISPR-based genome editing. (3) The scale mentions the year in which each (major) CRISPR tool was developed and used in agriculture.

Plants are exposed to different environmental stresses such as microbes or climatic changes which are referred to as biotic and abiotic stresses, respectively. Both of these (biotic and abiotic) cause almost 50% yield loss globally (Thabet and Alqudah, 2019; Alqudah et al., 2020). The forthcoming discussion presents a brief account of CRISPR application in cultivated grasses with some recent examples listed in Figure 2 and Table 1 and its potential to increase the quality as well as to combat the losses caused by stress.

**TABLE 1:** Application of CRISPR associated genome editing in major cultivated grasses.

	Crop	Gene	Function	References
Abiotic Stress	Wheat	*TaDREB2*	Drought tolerance	Bhowmik et al. (2018)
	Rice	*OsMYB1*	Tolerance to various environmental stresses	Mao et al. (2013)
	Barley	*HvPM19*	Regulator of grain dormancy under stress	Lawrenson et al. (2015)
	Maize	*ZmHKT1*	Salt tolerance	Xing et al. (2014)
	Barley	*inositol-tetrakisphosphate 1-kinase*	Tolerance to salinity stress	VlckoOhnoutkova (2020)
	Rice	*OsARM1 and OsNramp5*	Heavy metal (cadmium and arsenic) resistance	Tang et al. (2017)
	Rice	*OsPYL*	Enhanced high-temperature tolerance	Miao et al. (2018)
Biotic stress	Wheat	*TaABCC6 ABC*	ABC transporter (ABCC6) associated with Fusarium head blight (FHB) susceptibility	Cui. (2017)
	Rice	*OsSWEET11*	Resistance against pathogens and enhance yield	Xing et al. (2014)
	Sorghum	*DsRED2*	Biotic and abiotic stresses	Xing et al. (2014)
	Barley	*HvMORC6a*	Oomycetes resistance	Galli et al. (2022)
	Rice	*OsWRKY93 and OsMORE1a*	Resistant to viral diseases such as tungro disease and fungal disease (Magnaporthe oryzae)	Macovei et al. (2018), Kim et al. (2022), Li et al. (2021)
	Maize	*ALS*	Herbicide resistance	Svitashev et al. (2015)
	Barley	*HvMORC1*	Resistance against *Fusarium graminearum*	Kumar et al. (2018)
	Rice	*eif4g*	Resistant to tungro disease	Macovei et al. (2018)
	Wheat	*TaNFXL1*	Resistance against *Fusarium graminearum*	Brauer et al. (2020)
Quality Yield	Wheat	*Alpha-gliadin*	Gluten protein	Brandt et al. (2017)
	Barley	*GST* and *IPI*	Recombinant protein accumulation	Panting et al. (2021)
	Maize	*ZmIPK*	Phytic acid biosynthesis	Liang et al. (2014), Svitashev et al. (2015)
	Sorghum	*Alpha-kafirin*	Improving lysine and digestibility in sorghum	Li et al. (2018a)
	Rice	*SBEIIb*	High levels of amylose content	Sun et al. (2017)
	Maize	*Wx1*	Higher yield	Waltz. (2016)
	Barley	*HcCKK1*	Higher number of grains	Holubova et al. (2018)
	Barley	*HvCKX1*	Convert hulled into naked grains leading to higher grain yield, improved brewing quality	(Gasparis et al. (2018), Meints and Hayes (2019)
	Maize	*LIG, MS26, MS45*	Male-sterility	Svitashev et al. (2015)

### Abiotic Stress Resistance

It is important to note that abiotic stresses, such as drought (water shortage), flooding (hypoxia), salinity, heavy metals, temperature (hot and cold), and their interactions are the major factors hindering agricultural production. Using a variety of breeding approaches, different genes/pathways and regulatory networks involved in stress responses have been determined. To epitome, CRISPR-based genome editing has broadened the target of the biologists to activate or suppress the targeted genes involved in plant abiotic stress resistance (Osakabe et al., 2016). For example, heavy metal (cadmium and arsenic) resistance in rice plants was developed by knocking out *OsARM1* and *OsNramp5* genes using CRISPR-based gene editing (Tang et al., 2017; Wang et al., 2017). In wheat, two drought-associated genes, *TaDREB2* (dehydration responsive element binding protein 2) and *TaERF3* (ethylene-responsive factor 3) have been successfully targeted in the protoplast using CRISPR-Cas (Kim et al., 2018). Similarly, in comparison with wild-type, rice with CRISPR-Cas editing of the OsPYL abscisic acid receptor gene family showed enhanced high-temperature tolerance (Miao et al., 2018). An elevated expression of ARGOS8 (an auxin-related gene involved in organ size 8) in maize plants using CRISPR, helped to improve the drought tolerance in maize (Shi et al., 2017). Likewise, barley itpk1 (inositol-tetrakisphosphate 1-kinase) mutants were developed using a CRISPR-based genome editing tool to validate the gene function of *HvITPK1*, and insertion mutant lines revealed a higher tolerance to salinity stress than deletion mutants (Vlcko and Ohnoutkova, 2020). CRISPR-Cas 9-mediated *OsRR22* gene editing was applied to improve salt stress tolerance in rice (Zhang et al., 2019). Based on previously reported studies, precise base editing is one of the most suitable techniques which can be employed to develop mutants with loss or gain of function to develop stress-tolerant varieties. Similarly, some reports revealed the regulation of abiotic stress in plants by cis-regulatory sequences (Liu et al., 2014), so novel promoter variants can also be created to produce useful novel phenotypic variation and new quantitative trait loci (QTLs) by ‘gain-of-function’ mutation for various traits associated with abiotic stress tolerance.

### Biotic Stress Resistance

Plants are affected to varying degrees by biotic stress, among other environmental stresses. It has been possible with CRISPR-Cas -based genome editing to engineer crops resistant to bacterial, fungal, and viral diseases as well as oomycetes. For instance, rice, wheat, maize, and barley have seen great success in the ability to increasing resistance to powdery mildews, bacterial blights, and blast diseases (Chen et al., 2019; Mushtaq et al., 2021; Ali et al., 2022). A study conducted by Wang et al. (2014) developed a powdery mildew resistant wheat by disrupting the *TAMLOA1*, *TAMLOA2*, and *TAMLOA3* genes in the wheat genome using the CRISPR-Cas 9 system. In barley plants, the HvMORC1 gene is silenced via CRISPR-Cas 9 which led to an increase in the resistance against *Fusarium graminearum* (Kumar et al., 2018). Recently, CRISPR-mediated genome editing of the TaNFXL1 (Resistant; R) gene in wheat led to the enhanced resistance against *Fusarium graminearum* (Brauer et al., 2020). CRISPR-mediated genome engineering has also helped to produce eif4g rice that is resistant to viral diseases such as tungro disease (Macovei et al., 2018) and fungal disease (Magnaporthe oryzae) by genetic functional validation of OsWRKY93 and OsMORE1a gene (Li et al., 2021; Kim et al., 2022). A study about oomycetes resistance in barley investigated the functional genomics of *HvMORC6a* gene in barley by using the application of CRISPR technology (Galli et al., 2022). Although, genome editing has excelled with the application of CRISPR-based tools. But, there is still a huge gap to create disease-resistant germplasm which could be easier by targeting R and S-genes as well as their orthologues in other species. The optimization and reprogramming of CRISPR components could be useful to establish resistance against such biotic stresses for which no natural resistance is found.

### Improving Quality and Yield

Until now, the use of genome editing has had a positive effect on improving quality attributes such as starch content, fragrance, nutritional value, and storage durability in crop plants. The CRISPR-Cas9 system has been used to modify the starch branching enzyme gene SBEIIb to develop rice with high levels of amylose content with such nutritional properties of starch that benefit patients suffering from noninfectious chronic diseases related to diet and carbohydrates. (Sun et al., 2017). New ways of altering traits regulated by large, redundant gene families are being explored by CRISPR-Cas9 e.g., α-gliadin gene family, the major gluten encoding gene family in wheat that consists of more than 100 genes. Researchers have created low-gluten wheat by simultaneously knocking out the most conserved domains of α-gliadin family members that ultimately help to avoid celiac disease (Sanchez-Leon et al., 2018).

As a result of CRISPR-Cas technology, a company in the United States was able to mutate the waxy gene Wx1 to give higher yield maize for commercial use (Waltz 2016). In barley, the role of the cytokinin dehydrogenase enzyme (CKX1) was explored by silencing the HcCKK1 gene through CRISPR-Cas 9 which resulted in a higher number of grains in transgenic lines (Holubova et al., 2018). Similarly, CRISPR-mediated genome editing enabled the researchers to convert hulled into naked barley grains leading to higher grain yield and improved brewing quality (Gasparis et al., 2018; Meints and Hayes, 2019). The expression level of pro-nutritional phytase was modulated by targeting the barley PAPhy_a promoter (Holme et al., 2017). In cultivated grasses, many studies reported that the presence of various compounds with anticancer, anti-inflammatory, and anti-microbial properties such as anthocyanin in rice promotes growth and enhances environmental stress (Mackon et al., 2021), and lunasin peptide in barley, wheat and rye seeds possess chemo-preventive properties (Hernandez-Ledesmade et al., 2008; Nakurte et al., 2013). The application of CRISPR/Cas technology can be employed to target transcription factors regulating such compounds leading to quality improvement as well as yield.

## CRISPR-Cas Based miRNA-Editing for Crop Improvement

Micro RNA (miRNA) are small RNA molecules with complementary binding sites in target mRNAs, representing a promising avenue to control complex traits since miRNAs can precisely down-regulate any number of co-expressed target transcripts and their respective pathways (Tang and Chu, 2017). Interestingly, most of the miRNA-targeted transcripts encode transcription factors (TFs), which themselves often act as crucial hubs of developmental regulation. It should be noted that using SpRY, knocking out miRNAs regulated genes or engineering quantitative trait variation can be more efficient than Cas9 (Zhou et al., 2017). Recent research has revealed key functions for miRNAs in controlling crop plant agronomic traits including cereals. For instance, in rice, increased expression of the miR156-targeted *OsSPL14* is associated with improved grain yield, characterized by reduced vegetative branching (tillering) and increased panicle branching (Jiao et al., 2010; Miura et al., 2010). Further, the free threshing trait in wheat is caused by SNPs in the miRNA172 binding site of the major domestication gene Q (Liu et al., 2018). Taken together, integrating knowledge about miRNAs with advanced cereal molecular genetics techniques like CRISPR is a promising strategy for crop improvement. The small sequence size of miRNA sites makes loss-of-function mutation production is difficult to achieve. Thus, precise mutation developed by CRISPR-Cas9 is an excellent option to knockout miRNA small sequence in rice (Basso et al., 2019; Bi et al., 2020). Moreover, targeting the sequence prior to the miRNA region by CRISPR-Cas maximizes the chance of miRNA knockout. Chung et al. (2020) demonstrated that such an approach to creating a deletion within the pre-miRNA regions by CRISPR-rCas9 was efficient in knockout miRNAs in rice. Therefore, CRISPR can be applied to elucidate the function of miRNA-regulated genes in cultivated grasses and their relevance towards the improvement of agronomic traits. Thus, based on the given proofs-of-concept, we propose broadening the scope of CRISPR in miRNA-regulated genes in cultivated grasses for a deeper understanding of their function and role in crop improvement.

## Conclusion and Future Perspective

CRISPR-Cas represents the most recent development in genome engineering which has revolutionized crop breeding since 2013. With CRISPR-Cas, genome editing has become a relatively simple, low-cost, and robust process, resulting in huge advances in crop improvement.

### Regulation of GMOs

Producing disease-resistant and environment-adapted crops, as well as improving yields and quality, are the main applications of CRISPR technology in agriculture. One noteworthy example is that preassembled CRISPR-Cas9 ribonucleoproteins were delivered DNA-free into plant protoplasts of rice and wheat (Woo et al., 2015; Zhang et al., 2016). Thus, crops developed by DNA-free technology may be considered non-GM crops in some countries where GM crops are not allowed or are under prohibitive approval requirements. This would allow the development of higher-quality grains with better phenotypes that could be commercialized and sold. The European Union, however, does not permit ribonucleoprotein mutated crops without GMO approval (Gelinsky and Hilbeck, 2018).

### Well Suited Cas9 and SpRY Options

CRISPR/Cas as a powerful tool led to tremendous advances in crop improvement through precise knockout, knock-in, replacement, point mutations, and fine-tuning of any gene. A potential method to attenuate on-target editing and circumvent the vector self-editing, which gives rise to in activation, off-targeting, should be further explored with a clearer understanding of the characteristics underlying SpRY. Although Cas9 is not capable of PAM-less editing, however, it proved itself better than SpRY for canonical NGG PAM site editing, which reveals the unterminated importance of Cas9 (Ren et al., 2021). To date, SpRY is a choice for more fine exploration of plant genome and its application in rice plants will be rolled as the first confident presentation of unconstrained targeting with nearly PAM-less editing in monocots (rice) and dicots also as Ren et al. (2021) proved that SpRY can be used in Dahurian larch. It will inspire many exciting investigations such as the expansion of *in vivo* directed evolution efforts to improve other plant characteristics against resistance for high yielding of important agronomic traits to ensure sustainable food security.
